# Self-rated health and its related influencing factors among emergency department physicians: a national cross-sectional study

**DOI:** 10.3389/fpubh.2023.1147403

**Published:** 2023-07-13

**Authors:** Ke Peng, Jingjing Jiang, Nan Jiang, Rongrong An, Jianwei Zheng, Shijiao Yan

**Affiliations:** ^1^Department of Finance, Southampton Business School, University of Southampton, Southampton, United Kingdom; ^2^Department of Obstetrics and Gynecology, Tongji Hospital, Tongji Medical College, Huazhong University of Science and Technology, Wuhan, China; ^3^Department of Social Medicine and Health Management, School of Public Health, Tongji Medical College, Huazhong University of Science and Technology, Wuhan, China; ^4^Department of Biliary-Pancreatic Surgery, Tongji Hospital, Tongji Medical College, Huazhong University of Science and Technology, Wuhan, China; ^5^School of Public Health, Hainan Medical University, Haikou, Hainan, China; ^6^Key Laboratory of Emergency and Trauma of Ministry of Education, Hainan Medical University, Haikou, China; ^7^Department of Emergency Medicine, Hunan Provincial Key Laboratory of Emergency and Critical Care Metabolomics, Hunan Provincial Institute of Emergency Medicine, Hunan Provincial People's Hospital/The First Affiliated Hospital, Hunan Normal University, Changsha, China

**Keywords:** self-rated health, emergency department, physicians, occupational health, effort-reward imbalance

## Abstract

**Background:**

Protecting and improving the personal health of healthcare workers is critical to improving the efficiency and quality of care. To effectively meet the needs of the emergency service system, emergency physicians need to be in a good state of health. However, due to the special characteristics of work in the emergency department, emergency physicians have to face various psychosocial pressures, which may bring them physical and mental distress. Therefore, this study aims to explore the emergency physicians' self-rated health status and its related factors, to provide an empirical study for the improvement of emergency physicians' self-rated health status.

**Method:**

A cross-sectional survey of emergency physicians was conducted in China between July and August 2018. The questionnaires contained items on demographic characteristics, behavioral lifestyle and job-related factors, as well as self-rated health. The generalized ordinal logistic model was used to identify related factors of emergency physicians' self-rated health status.

**Results:**

Only 14.4% of Chinese emergency physicians considered themselves in good health status. Results showed that emergency physicians who never exercised (β = 0.76, *p* < 0.001) and exercised <2 times per week (β = 0.34, *p* < 0.001) were more likely to report poor health status. In addition, emergency physicians with good sleep quality (β = −3.84, *p* < 0.001), fewer night work shifts (β = −0.47, *p* < 0.001), less frequency of visiting patients (β = −0.33, *p* < 0.001), never suffered the workplace violence (β = −0.47, *p* < 0.001) and never perceived effort-reward imbalance (β = −0.68, *p* < 0.001) were more likely to report good self-rated health.

**Conclusion:**

Chinese emergency physicians' self-rated health status was not optimistic. Self-rated health is associated with multiple domains of work-related factors and personal lifestyle. Feasible measures should be taken to improve the working environment of emergency physicians, develop acceptable shift schedules for employees, monitor and maintain the health status of emergency department physicians.

## Introduction

The World Health Organization emphasizes that ensuring and improving the health of physicians globally is essential to effectively respond to the health care needs of the population (1). NHS data indicate that poor health of medical staff may affect the efficiency of treatment and quality of care (2). As the first line of defense in the healthcare system, emergency departments accept most patients who are seriously injured or critically ill, so emergency physicians need to quickly evaluate the clinical situation of patients and take action (3). But unhealthy emotional state will affect doctors' work decisions and relationships with patients (4–6). In addition, in the context of a shortage of human resources in emergency medicine (7), the good health status of emergency physicians is also crucial to effectively respond to the increasing demand for emergency services. Therefore, it is necessary to explore the health status of emergency doctors and identify its related factors.

Self-rated health is a more inclusive measure of health, covering a wider range of physical and mental health (8). This indicator reflects the current state of the human body, which depends on the difference between the individual's expectations of health status and the rating scale (9). Self-rated health status is now widely used as an indicator of the comprehensive health status of health care professionals (10, 11), and most previous studies have focused on primary healthcare works, residents and medical students. The study showed that 78.8% of the resident physicians in Finland rated themselves as good (12). The rates of self-rated good health status of Norwegian general practitioners and German hospital doctors were 88.1% and 63.3% (13), and 78% of primary care workers in Brazil rated themselves as in good health (14). Previous studies on the self-rated health status of physicians have shown that in addition to gender and age, the related factors of self-rated health status are divided into lifestyle and work environment, including obesity, irregular working hours, workplace violence, and effort-reward imbalance (15–18). But this association has been less validated among emergency physicians. Only one study from South Korea showed that emergency physicians rated their health as poor, which was related to night shift and mood (19). Considering that the national emergency medical system and social economic development are at different stages, emergency departments have great differences in the working environment, patient groups and other aspects. Therefore, it is necessary to conduct research on the self-rated health status of emergency physicians and its related factors in China.

As one of the largest number of emergency patients receives country in the world every year, overcrowding in Chinese emergency departments is very common in all level hospitals (20). In addition, the number of emergency medical visits in China has increased sharply in recent years, which makes the receiving task of the limited emergency doctors more and more heavy, and the hospitalization time of patients is longer, resulting in serious mental and physical exhaustion of the emergency labor force (21–24). In the context of the mismatch between the demand for emergency services and human resources in emergency departments in China (25), it is more necessary for emergency physicians to have good health to improve their efficiency (26, 27). To our knowledge, there are no studies on self-rated health status and its related factors among Chinese emergency physicians. Thus, this large cross-sectional study was conducted among emergency physicians in China, aiming to understand the prevalence of self-rated health and explore its associated factors among emergency physicians, which would provide an empirical basis for the improvement of self-rated health among emergency physicians.

## Method

### Ethics statement

This study was approved by the Medical Ethics Committee of Hainan Medical College (HYLL-2018-035). The purpose of the study was explained to all participants prior to the survey, the study was voluntary and anonymous, and all questionnaire information was used for scientific research only.

### Study design and data collection

This study was part of a national cross-sectional survey of emergency medical resources conducted in China, with the coordination of the National Medical Administration Bureau of the National Health Commission of the People's Republic of China, from July to August 2018. An anonymous questionnaire with standard structure was used to collect data through online survey platform in China (platform name: Questionnaire Star Project, at https://www.wjx.cn). The web-based questionnaire link was distributed to the emergency department physician work platform of the pre-hospital emergency facility configuration monitoring department, inviting emergency department physicians to participate anonymously in this cross-sectional online survey. Additionally, the link of the questionnaire was reposted to the platform every 7 days to remind emergency physicians to complete the anonymous survey until it ended. All participants were required to read and agree to an electronic version of the informed consent statement before they could complete the survey by visiting the link. The data were stored and administered by the Questionnaire Star platform.

In this study, we included physicians who worked in emergency department and volunteered to participate in the survey and excluded those interns who had not yet obtained a practicing certificate. A total of 15,288 emergency department physicians clicked on the survey link during the study period, of which 10,457 completed the questionnaire. The response rate was 68.4%.

### Quality control

This study mainly adopts two quality control measures to ensure the quality of online question questionnaire results. First, to prevent duplication of responses, each mobile phone number can only be used once for a complete questionnaire. Second, we set three quality control questions at different places of questionnaire. The online questionnaire system would mark the questionnaire as invalid if incorrect answers to the quality control questions appeared.

### Measurements

Data were collected using a standard questionnaire developed based on a review of published literature. A pilot study including 30 physicians was conducted before the formal survey to pretest that the questions were clear and easy-to-understand to all participants.

The items of this questionnaire covered socio-demographic characteristics, behavior lifestyles, work-related factors and the outcome variable of self-rated health. Specifically, socio-demographic characteristics included sex, age and job seniority. Behavior lifestyles included physical exercise frequency per week and sleep quality. Work-related factors comprised night shift frequency per month, number of patients seen by the physicians per day, and whether emergency department nurses experienced workplace violence in the past year, as well as perceived effort-reward imbalance.

### Outcome

In this study, self-rated health of emergency physicians was investigated using an item: “How do you think your health has been in the past 6 months?” To date, various studies have identified self-rated health using similar questions with three to five response options (11, 12, 27). The results of this study were analyzed using a 5-point Likert-type scale (very good = 1, good = 2, normal = 3, poor = 4, very poor = 5). Higher scores indicate poorer physician-reported health status. To simplify the results of the study, the self-rated health was divided into three groups based on previous studies, with emergency doctors scoring 1 and 2 being classified as good, 3 as normal and 4 and 5 as poor.

### Behavior lifestyles

The frequency of exercise was measured by a question that asked, “In the past 6 months, The number of times per week you exercise for more than 30 min”. The respondents' sleep status was measured using a question “In the last 6 months, please rate how well you slept at night.” Responses were assessed using a 5-point Likert scale (very good = 1, good = 2, normal = 3, poor = 4, very poor = 5). To simplify the analysis, the sleep status of emergency physicians was divided into three groups, with those scoring 1 and 2 being categorized as good sleep quality, those scoring 3 as normal sleep quality and those scoring 4 and 5 as poor sleep quality.

### Work-related factors

Exposure to workplace violence was measured by two questions: “Have patients verbally (physically) assaulted you while you were at work in the past year?” The results were answered with “yes” and “no”. The exposure of emergency physicians to workplace verbal violence and workplace physical violence is shown separately.

The balance between work effort and reward was assessed by the Effort-Reward Ratio (ERR), which was measured using a subscale of the Effort-Reward Imbalance Questionnaire (ERI) (28, 29). The effort consists of six items scored on a 5-point Likert scale (from 1 = strongly disagree to 5 = strongly agree), with higher scores associated with greater effort, and the reward consists of 11 items scored on a 5-point Likert scale (from 1 = strongly agree to 5 = strongly disagree), with higher scores associated with greater perceived reward. The effort-reward ratio is calculated as follows: ERR = (11 × effort)/ (6 × rewards). Therefore, an ERR > 1.0 indicates that the effort put in is not adequately rewarded. (The Cronbach's α for effort and reward is 0.82 and 0.92 respectively).

### Statistical analysis

All data analyses were conducted using the Social Science (SPSS) version 23 (SPSS Inc., Chicago, IL, USA). For descriptive analyses of respondent characteristics and self-rated health status, categorical variables were expressed using frequencies and percentages, and continuous variables were described using means and standard deviations. A chi-square test was conducted to describe sociodemographic characteristics and work-related factors in different self-rated health groups among physicians in emergency department. Multicollinearity of the independent variables was tested by calculating the variance inflation factor (VIF) (min =1.04, max=2.01). A variance inflation factor <10 indicated that no covariance was detected. Considering that outcome variables were ordered categorical variables and did not satisfy normal distribution, we used Generalized ordered logistic regression analysis to identify influencing factors of self-rated health status. Generalized ordered logistic regression model compares all the categories greater than the current category to those less than or equal to the current category. Hence, positive coefficients indicate that higher values of the explanatory variable increase the likelihood of the respondent being at a higher health level than at the current or lower health levels, whereas negative coefficients indicated that higher values of the explanatory variable increase the likelihood of the respondent being at the current or lower health levels than at a higher health level. The level of significance or α was set at 0.05 for all statistical tests.

## Result

Descriptive statistics of the participants are shown in Table 1. Among the 10,457 emergency physicians, the majority of participants were male (73.0%), most of them were between the ages of 17 and 44 (82.8%), only 10.4% had a good sleep profile and 46.8% had not been physically active in the last 6 months. Overall, more than half of the emergency physicians worked between 6 and 10 nights shifts per month (53.9%), and 41.4% received between 1 and 10 visits per day.

**Table 1:** Characteristic of physicians.

**Variables**	**All subjects (*N*, %)**	**Good Self-rated health (*N*, %)**	**Normal Self-rated health (*N*, %)**	**Bad Self-rated health (*N*, %)**	**χ^2^**	***P*-value**
**Total**	10,457	1,499 (14.3)	5,130 (49.1)	3,828 (36.6)		
**Gender**					23.21	<0.001
Male	7,632 (73.0)	1,078 (71.9)	3,628 (70.7)	2,926 (76.4)		
female	2,825 (27.0)	421 (28.1)	1,502 (29.3)	902 (23.6)		
**Age**					47.22	<0.001
≤ 30	2,600 (24.9)	469 (31.3)	1,309 (25.5)	822 (21.5)		
31–40	4,977 (47.6)	552 (36.8)	2,410 (47.0)	2,015 (52.6)		
41–50	2,388 (22.8)	366 (24.4)	1,156 (22.5)	866 (22.6)		
≥51	492 (4.7)	469 (31.3)	1,309 (25.5)	822 (21.5)		
**Work year**					41.57	<0.001
<1	1,448 (13.8)	281 (18.7)	789 (15.4)	378 (9.9)		
1–5	3,965 (37.9)	591 (39.4)	1,971 (38.4)	1,403 (36.7)		
6–10	2,458 (23.5)	280 (18.7)	1,146 (22.3)	1,032 (27.0)		
≥11	2,586 (24.7)	347 (23.1)	1,224 (23.9)	1,015 (26.5)		
**Behavior lifestyle**						
**Physical exercise** (**time/week)**					325.21	<0.001
0	4,897 (46.8)	376 (25.1)	2,290 (44.6)	2,231 (58.3)		
1–2	3,748 (35.8)	606 (40.4)	1,952 (38.1)	1,190 (31.1)		
≥3	1,812 (17.3)	517 (34.5)	888 (17.3)	407 (10.6)		
**Sleep quality**					2,715.21	<0.001
Good	1,087 (10.4)	732 (48.8)	306 (6.0)	49 (1.3)		
Normal	3,227 (30.9)	568 (37.9)	2,326 (45.3)	333 (8.7)		
Bad	6,143 (58.7)	199 (13.3)	2,498 (48.7)	3,446 (90.0)		
**Work-related factor**						
**Night work** (**time)**					175.50	<0.001
≤ 5	2,033 (19.4)	522 (34.8)	1,105 (21.5)	406 (10.6)		
6–10	5,633 (53.9)	705 (47.0)	2,814 (54.9)	2,114 (55.2)		
11–15	2,252 (21.5)	223 (14.9)	999 (19.5)	1,030 (26.9)		
≥16	539 (5.2)	49 (3.3)	212 (4.1)	278 (7.3)		
**Visit patient**					53.01	<0.001
1–10	4,333 (41.4)	766 (51.1)	2,198 (42.8)	1,369 (35.8)		
11–20	2,202 (21.1)	319 (21.3)	1,080 (21.1)	803 (21.0)		
21–30	1,547 (14.8)	176 (11.7)	787 (15.3)	584 (15.3)		
≥31	2,375 (22.7)	238 (15.9)	1,065 (20.8)	1,072 (28.0)		
**Experienced verbal violence in the past year**					877.89	<0.001
No	1,902 (18.2)	631 (42.1)	987 (19.2)	284 (7.4)		
Yes	8,555 (81.8)	868 (57.9)	4,143 (80.8)	3,544 (92.6)		
**Experienced physical violence in the past year**					530.82	<0.001
No	7,568(72.4)	1,273 (84.9)	4,020 (78.4)	2,275 (59.4)		
Yes	2,889 (27.6)	226 (15.1)	1,110 (21.6)	1,553 (40.6)		
**ERR**					882.89	<0.001
≤ 1	2,260 (21.6)	659 (44.0)	1,286 (25.1)	315 (8.2)		
>1	8,197 (78.4)	840 (56.0)	3,844 (74.9)	3,513 (91.8)		

Table 1 shows that only 14.3% of emergency physicians rated their health as good, and nearly half of them rated their health as normal (49.1%). There were 78.4% of emergency physicians had an ERR >1.0, which represents an effort-reward imbalance situation. Univariate analysis showed significant differences among the group in age, gender, frequency of exercise, sleep quality, years of work, frequency of night shifts, number of consultations, effort-reward imbalance, and workplace violence (*p* < 0.001).

Table 2 showed the results of analyses of generalized ordinal logistic regression models of self-rated health. Regarding the personal factors, emergency physicians who did not engage in physical activity for the last 6 months (β = 0.75, *p* < 0.001) and exercised less than twice a week (β = 0.34, *p* < 0.001) reported poorer health. Physicians with good sleep quality (β = −3.84, *p* < 0.001) and normal sleep quality (β = −1.88, *p* < 0.001) reported better self-rated health. Regarding work-related factors, doctors with <1 year of emergency work (β = −0.18, *p* = 0.017) and those with 1–5 years of work (β = −0.20, *p* < 0.001) perceived their health good. Emergency physicians with <5-night shifts per month self-rated their health as better (β = −0.48, *p* < 0.001). Physicians with <10 frequent visits (β = −0.33, *p* < 0.001) were less likely to perceive poor health compared to physicians with >31 visits per day. In addition, Emergency physicians who had not experienced workplace violence (β = −0.47, *p* < 0.001) in the past year were more likely to report good self-rated health. Similarly, doctors who did not perceive the imbalance between reward and effort (β = −0.69, *p* < 0.001) were also more likely to self-rate good health.

**Table 2:** Generalized ordinal logistic model examining factors associated with the self-rated health.

**Variables**	**Coefficient**	**SE**	***P*-value**	**(95%CI)**	
**Sex (Ref:female)**				**Lower limit**	**Upper limit**
Male	−0.09	0.05	0.049	−0.19	0.00
**Physical exercise(time/week) (Ref:**>**3)**					
0	0.75	0.06	<0.001	0.63	0.87
1–2	0.34	0.06	<0.001	0.22	0.46
**Sleep quality (Ref:Bad)**					
Good	−3.84	0.09	<0.001	−4.01	−3.67
Normal	−1.88	0.05	<0.001	−1.99	−1.77
**Work year (Ref:**>**11)**					
<1	−0.18	0.07	0.011	−0.32	−0.04
1–5	−0.20	0.05	<0.001	−0.30	−0.09
6–10	−0.05	0.06	0.399	−0.17	0.07
**Night work(time)(Ref:**>**16)**					
≤ 5	−0.48	0.10	<0.001	−0.68	−0.27
6–10	−0.20	0.10	0.036	−0.39	−0.01
11–15	−0.15	0.10	0.131	–−0.35	0.05
**Visit patient (Ref:**>**31)**					
1–10	−0.33	0.05	<0.001	−0.44	−0.22
11–20	−0.18	0.06	0.005	−0.30	−0.05
21–30	−0.18	0.07	0.008	−0.32	−0.05
**Experienced verbal violence in the past year (Ref:Yes)**					
No	−0.47	0.06	<0.001	−0.59	−0.36
**Experienced physical violence in the past year (Ref:Yes)**					
No	−0.46	0.05	<0.001	−0.55	−0.36
**ERR(Ref:**>**1)**					
≤ 1	−0.69	0.05	<0.001	−0.795	−0.585

Deviance test *p* = 0.882 > 0.05.

## Discussion

This study aimed to understand the prevalence of self-rated health and explore its associated factors among emergency physicians. This is the first study to explore factors associated with self-rated health among a national population of emergency physicians in China. This study is helpful to better understand the health status of emergency physicians in China and provide representative Chinese data for understanding the self-rated health level of emergency physicians around the world.

The results show that only 14.3% of emergency physicians in China consider themselves in good health, which is lower than the 40.1% of healthcare professionals in other departments in China who report being in good health (27). In addition, the rate of emergency physicians reporting better health was significantly lower in China compared to the self-rated health status of Norwegian physicians (88.1%) (11), Swiss primary care physicians (94%) (5), German physicians (63.3%) (13) and Lithuanian physicians (61.6%) (17). This difference may be due to the greater workload and unpredictability of emergency physicians compared to other health workers (22). They are chronically exposed to occupational stresses such as rapid decision-making, overcrowding, resource shortages and exposure to traumatic events (30). These challenges in emergency department work are more likely to be mentally and physically burden for emergency physicians. Due to the lack of special researches on the health status of emergency physicians at home and abroad, it is difficult to compare the self-rated health status of emergency physicians among countries.

The results showed that the behavioral lifestyle of emergency physicians was related to their self-rated health status, and the emergency physicians with few physical activity and poor sleep quality were more likely to report poor self-rated health status. Previous studies of the general population have similarly shown that regular exercise habits have a positive impact on overall morbidity and mortality, as well as protection against many chronic diseases (12, 31). In our study, only 17.3% of physicians were physically active more than three times a week. Therefore, advocating weekly appropriate physical exercise among emergency physicians can be a beneficial measure for self-management of health (16). In addition, more than half of the doctors reported suffering from poor sleep status, possibly because the irregular working hours and shift schedules in emergency departments make it difficult for emergency physicians to relax after work and the constant mental stress makes them more likely to have poor sleep quality (12, 15, 17, 32). Chronic sleep disturbances can lead to dysregulated circadian rhythms, resulting in decreased vitality and increased burnout (33), which can burden physicians' emotional state and physical condition. Thus, hospital managers should pay attention to the quality of life of emergency physicians, strengthen the personal health education of emergency doctors, and promote active exercise for emergency physicians to relieve the stress of intense work through a combination of work and rest.

The results also showed that job-related factors were also significantly related to self-rated health. This study found that emergency physicians with a low frequency of night shifts per month and a low number of daily visiting patients reported better health status. Possible explanations are that increased clinical workloads and long hospital shifts can increase the physical strain on emergency physicians. Long work hours can lead to insufficient recovery, which in turn may cause various health problems (13). In addition, irregular working hours and frequent questions from patients and families can lead to physician burnout (34), which had a negative impact on self-rated health. Similar results have been reported among Norwegian physicians and Swedish health workers to explain the important effect of shift work and long working hours on self-rated health status. Therefore, in terms of health care policy, better time control may be an important measure to improve physical health, and hospital managers should reduce the risk of physician overwork by allocating human resources to the emergency department, and reducing the workload of on-call physicians (4).

The effort-reward imbalance model is the dominant model to explain work stress. In this study, 78.4% of emergency physicians experienced ERI. This result can be explained by the national conditions and the work characteristics of the medical system in China. The available resources of the Chinese healthcare staff cannot meet the needs of a large number of patients (26), which requires healthcare workers to expend more effort to achieve overall organizational goals (27). This study showed that effort-rewards imbalance had a negative impact on the self-rated health of emergency physicians. Accordingly, the same results were obtained in studies of other occupations (35–37). A possible reason is that imbalance between effort and reward (ERR > 1) may lead to a state of “active distress” by evoking strong negative emotions (28). This chronic emotional conflict can affect emergency physicians manage their relationships with colleagues and patients, making them more prone to a mental breakdown and physical harm (38, 39). In addition, strong negative emotions can cause emergency physicians to awaken stress-related reactions autonomously, and ERI can exacerbate the development of physical and mental illness in emergency physicians as emergency departments work under the pressure of dealing with unexpected situations throughout the year (40, 41). Therefore, managers need to consider the psychosocial work stress of healthcare workers, adjusting workloads and reward systems, and provide better compensation schemes to reduce the burden of health-related stress through individual and organizational development measures.

The results showed that workplace violence was associated with self-rated health status of emergency physicians. The possible reason is that workplace violence is an important occupational hazard and stressor for medical professionals (42). It can cause anger, anxiety, fear and depression among emergency physicians, posing a threat to their mental health. In our study, 27.6% of emergency doctors in China experienced physical violence and 81.8% of doctors experienced verbal violence, which is far higher than doctors in other departments in China (43). Therefore, given the multiple negative impacts of violence on the health of emergency physicians, emergency department managers should prevent workplace violence before it occurs and improve workplace systems. In addition to stabilizing the external working environment, immediate decisions should be made to help emergency physicians reduce the mental and physical distress caused by violence.

### Strengths and limitations

This is a cross-sectional study with a large sample size at the national level and is the first time to report the self-rated health status of emergency physicians and its associated factors. In addition, due to the large sample size and the distribution of participants across multiple provinces and cities, our findings have highly representative.

However, the limitations of this study should be considered. Firstly, this study relied on self-reported questionnaire data and there was some recall bias, which may have some impact on the accuracy of the results. Secondly, this study used cross-sectional data, which cannot explain the causal findings, and a longitudinal study is needed to further verify the causal relationships between the variables. Thirdly, in addition to the factors that were investigated in this study, other potential factors may be related to turnover intention and should be studied in future research.

## Conclusion

We found that the self-rated health status of Chinese emergency physicians was not optimistic, with only 14.4% of physicians considering themselves to be in good health. The findings suggest that exercise frequency, sleep status, years of experience, frequency of night shifts, number of consultations, effect-reward imbalance, and workplace violence are significantly associated with the self-rated health of emergency physicians. These results may help hospital managers to develop effective and sustained health interventions at a systemic level (including organizational, cultural, social and physical) to reduce the hazards of psychological and occupational stress among emergency physicians.

## Data availability statement

The raw data supporting the conclusions of this article will be made available by the authors, without undue reservation.

## Ethics statement

All the methods in this study were in accordance with the Institutional Research Committee and Tenets of the Declaration of Helsinki. All participants provided informed consent.

## Author contributions

KP, NJ, and SY designed this study and collected the data. KP and JJ completed data analysis and drafted the main manuscript text. NJ, JZ, and RA provided the critical revision of the manuscript and supervision and all the processes of the work. SY was responsible for the conception, design, and writing of the manuscript. All authors reviewed and approved the manuscript.
