# Erratum: Self-rated health and its related influencing factors among emergency department physicians: a national cross-sectional study

**DOI:** 10.3389/fpubh.2023.1273905

**Published:** 2023-08-15

**Authors:** Frontiers Production Office

**Affiliations:** Frontiers Media SA, Lausanne, Switzerland

**Keywords:** self-rated health, emergency department, physicians, occupational health, effort-reward imbalance

Due to a production error, a note stating that Ke Peng is the first author and Jingjing Jiang is the co-first author was omitted from the original article.

The publisher apologizes for this mistake. The original article has been updated.

